# Supplementary material for: Finite-size effects on bacterial population expansion under controlled flow conditions
Source: Sci Rep. 2017 Mar 6;7:43903. doi: 10.1038/srep43903 (PMC5338255; doi:10.1038/srep43903)
Supplement: Supporting Information [file srep43903-s1.pdf]

Supporting Information for "Finite-size effects on bacterial population expansion under controlled flow conditions"

Francesca Tesser, Jos C.H. Zeegers, Herman J.H. Clercx, Luc Brunsveld and Federico Toschi

Details on the experimental set-up, on the flow characterization and on the procedure to extract the front speed of the bacterial colony from the intensity profiles are described here.

Flow characterization

The values of the imposed flow velocity in each channel have been obtained from a Lattice Boltzmann simulation. The *15ch* device has been discretized into (501×440×14) lattice units (l.u.), which corresponds to 20 μm/l.u., while the *42ch* device into (950 ×475 ×7) l.u., which means 40 μm/l.u.. The magnitude of the velocity field at mid-height in the device is shown in Figure S2, where the data are normalized by the mean inflow velocity v_{in}. The simulations have been initialized by setting no-slip boundary conditions at the walls and inflow and outflow boundary conditions at the entrance and exit planes of the device. The centerline velocity in the channels in the *15ch* device is also plotted in Figure S3 for the typical inflow rate Q=0.1 μL/min.

This flow has been validated both by using the network analysis, fixing the input flow rate and solving the pressure drop at each fluidic node, and experimentally in the device using Particle Tracking Velocimetry technique (PTV) on polystyrene beads of radius 1.5 μm (PolyspherexTM). The PTV experiment has been performed at inflow rate Q=1 μL/min and the output has been analyzed using *Trackpy v0.3.0.* The resulting velocities are normalized by the inflow velocity in order to compare them with the other methods (see Figure S4).

Image Processing

The raw images acquired with fluorescent filters are processed and analyzed with Python. As general procedure, the noise outliers pixels are removed in the whole time stack, by replacing them with the median 2-pixel radius filter value, then the first image, characterized by no bacteria in the channels is subtracted to all images as background and rotation is applied, if needed.

Figure S5 shows the proportionality between the bacteria concentration, inside independent channels of cross section 300 μm × 280 μm, and the fluorescence intensity images collected by the camera and post-processed in the usual way. The points in the graph refer to the mean intensity per unit surface, measured from images of independent channels homogeneously filled by several dilutions of a reference bacterial concentration c_0. The x-axis spans a reasonably wide range of concentrations, similar to the ones of the experiment.

In Figure S6 three snapshots of the bacterial fluorescence intensity in the device are shown. The pixel intensity is usually integrated along the transversal direction of each channel in order to express the intensity profile only along the direction of growth; finally a Gaussian 3-pixel filter is applied to this one-dimensional signal to smooth it. At this stage we have a time series of bacterial density for each channel along the overall propagation direction. The front profile is usually disturbed by a background growth, which at late time is covering the signal. This growth is not affecting the measurement if we consider the front only at its initial stage. Operatively, we measure the front only before a maximum time value in combination with a cutoff on the signal on the x-axis, as shown in Figure S7 on the left. The front position can be determined by fixing a threshold on the signal intensity and its speed can be calculated as the slope of a regression line of the detected front positions over time. In order to make the speed estimate independent on the threshold choice when the front shape is not regular, we

proceed by fixing a set of incremental thresholds, between a minimum and maximum level, and averaging the corresponding set of slopes. This procedure is sketched in Figure S7. The mean slope, weighted by the linear correlation coefficient, which quantifies the quality of the linearity, is the extracted speed of the front and the standard deviation of the distribution of slopes is taken as the error. In this way bad choices of threshold are automatically neglected and more importance is given to the parts of the front which are propagating at constant speed.

Front width and maximum density

We quantify the intensity profiles in the channels corresponding to the experiments with Q=0.1 μL/min and integrate the fluorescence intensity in the part of the channel behind the front in the stationary regime. These values are plotted for four identical experiments as a function of the maximum velocity in the channels in Fig. S9, together with the mean value. We observe that the density is systematically higher for negative velocities, while the populations appear systematically diluted in the co-flow regime. Finally, also the front width has been estimated from the front profiles. The data come from a fit on the raw front profiles (no Gaussian filter is applied to the signals for this analysis) with an hyperbolic tangent function, f(x)=a[1-b\tanh{(cx+d)}]. The width corresponds to 2/c. The fits have been performed at each time value and the front width has been extracted at the stationary regime, which can also corresponds to slightly different time values from channel to channel. The width shown in Figure S10 appears to have a systematic variation with the flow direction and intensity, in particular to be larger in the co-flow regime.

1D hard beads model

Numerical data from the one-dimensional model have been obtained from the average over 20 independent realizations. For each realization the front speed has been calculated over 1200 generation times or, for strong counter-drift, over a length of around 1200r. Parameters of this simulations are D=0.0036, μ=0.5, r=0.088 and dt=0.05. The simulation is initialized always with three beads.

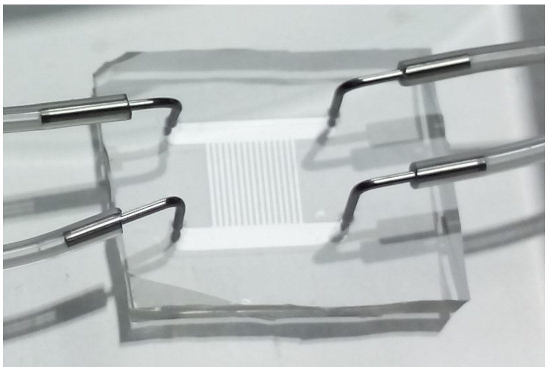

Figure S1: A picture of a microfluidic device composed of 15 channels with metal connectors for the external tubing system.

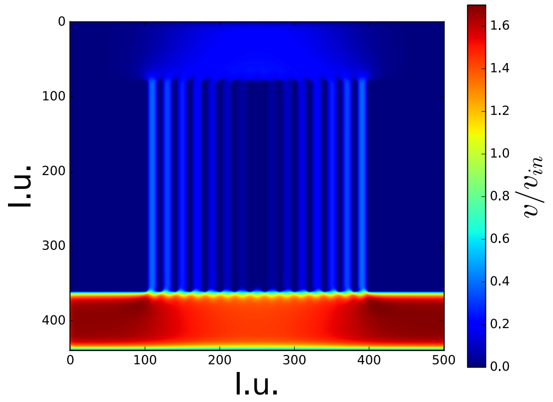

Figure S2: The dimensionless magnitude of the velocity in the mid-plane of the device obtained by LBM simulations normalized by the mean inflow velocity v_{in}. Size of the system is (501 × 440) l.u., with 1 l.u. corresponding to 20 μm.

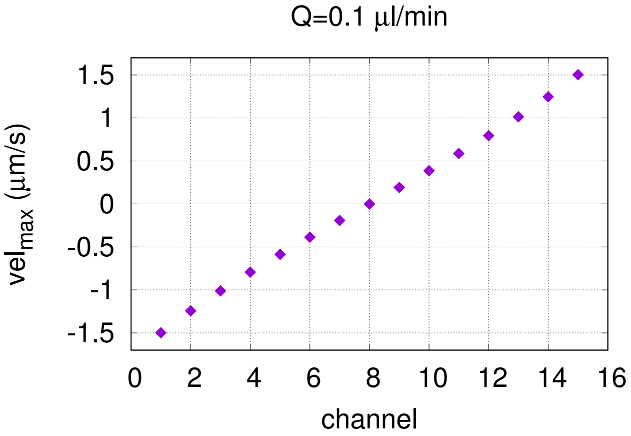

Figure S3: Maximum centerline velocity in the 15 channels with inflow rate Q=0.1 μL/min simulated with the Lattice Boltzmann Method.

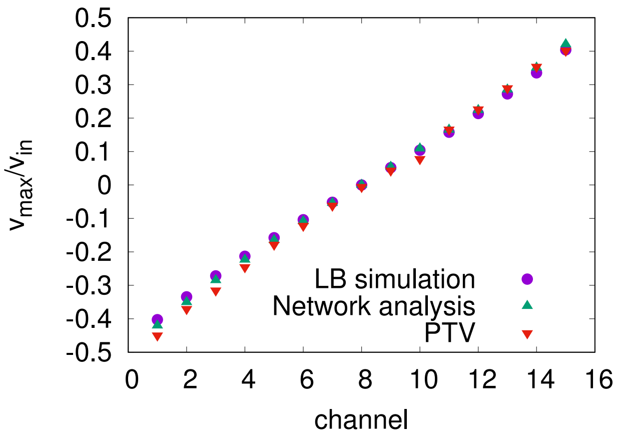

Figure S4: Maximum velocity in the channels of the *15ch* device normalized by the mean inflow velocity v_{in}. Comparison between the values obtained from a Lattice Boltzmann Simulation, the network analysis and PTV experiments on polystyrene beads.

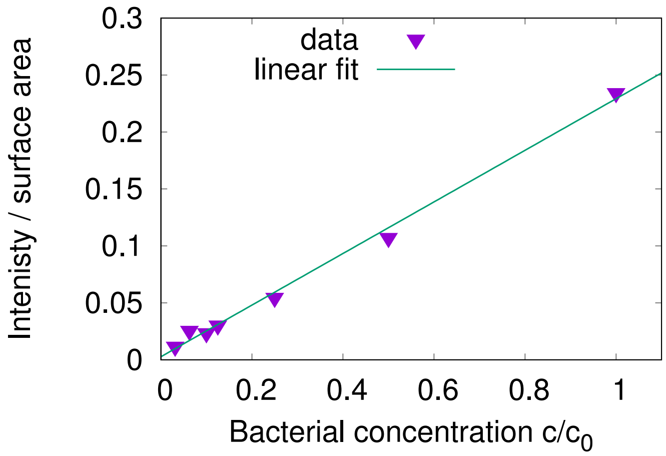

Figure S5: Proportionality between bacterial concentration and the fluorescent signal collected by the camera from independent channels.

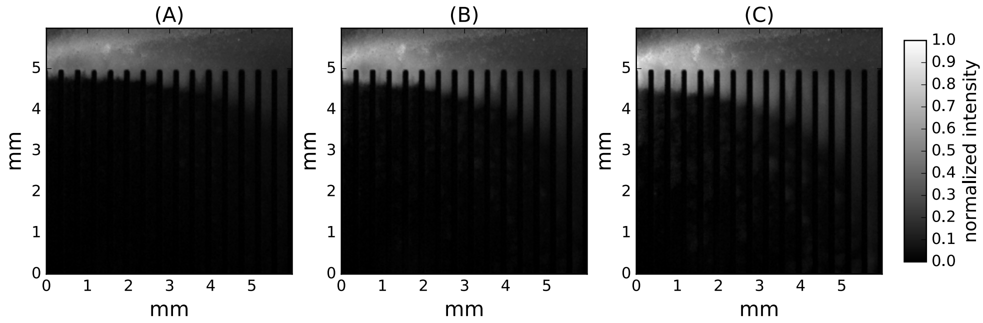

Figure S6: Three snapshots of fluorescent intensity in the 15ch device for an experiment with inflow Q=0.1 μL/min, corresponding to time t_1=5.7h, t_2=6.7h and t_3=7.6 hours from the beginning of the experiment. The data plotted here have also been filtered by a 3-pixel radius Gaussian filter. The initial colony of bacteria deposited in the upper part of the device grows along the channels experiencing different flow velocities.

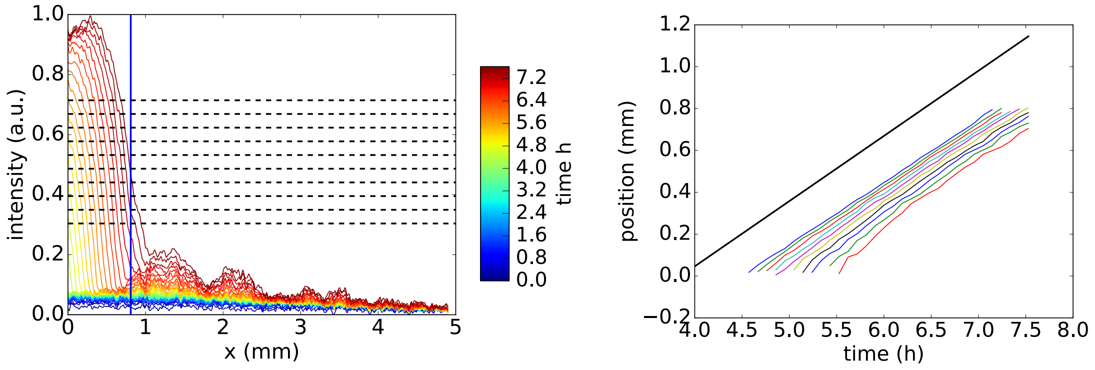

Figure S7: (Left) In order to determine the front speed, multiple intensity thresholds are chosen equally spaced between a minimum and a maximum level of signal intensity, in this plot only 10 representative dashed lines are sketch as example, while in general around 200 lines are considered. Each threshold intercepts the signal at a given time, at a specific position. The vertical line corresponds to the cutoff on the signal. Only the signal at the left of the cutoff is considered. (Right) For each threshold, the front position is plotted as a function of time and a regression line is extracted, together with its regression coefficient. The mean on the collected slopes, weighted by the regression coefficients, is the final value of speed attributed to the front and it corresponds in this example to the black line.

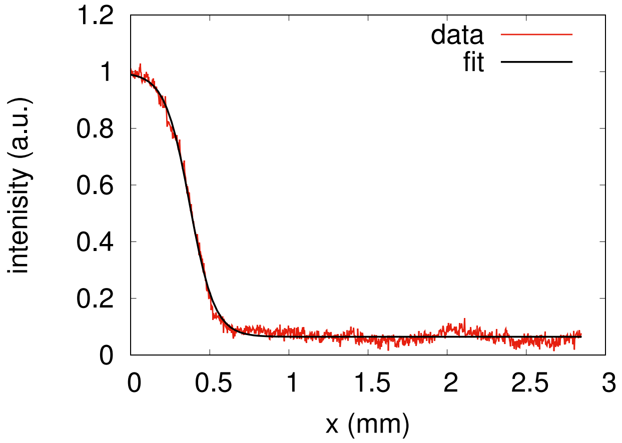

Figure S8: Fit of the raw front profile by a hyperbolic tangent on channel number 4.

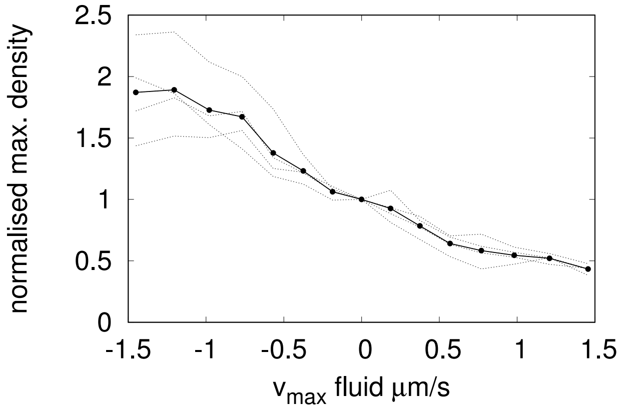

Figure S9: Maximum density of bacteria behind the front in the stationary regime as a function of maximum velocity in the channel, normalized to the maximum density in the zero-flow channel. Black dots are the mean values of four repeated single experiments (gray lines), which give an idea of the uncertainty.

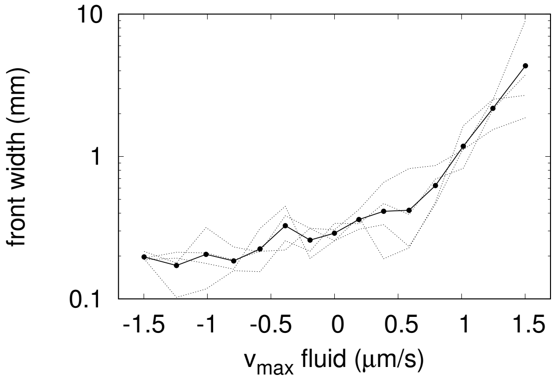

Figure S10: Front widths as a function of maximum velocity in the channel plotted in a logarithmic scale on y-axis. Gray lines refer to 4 identical experiments, while the black dots are the mean values.
